# Enhancing the nutritional value and antioxidant properties of foxtail millet by solid‐state fermentation with edible fungi

**DOI:** 10.1002/fsn3.4203

**Published:** 2024-07-01

**Authors:** Tong Lin, Zhanyong Li, Gongjian Fan, Chunyan Xie

**Affiliations:** ^1^ College of Life Science, Langfang Normal University Langfang Hebei People's Republic of China; ^2^ Technical Innovation Center for Utilization of Edible and Medicinal Fungi in Hebei Province Langfang Hebei People's Republic of China; ^3^ Edible and Medicinal Fungi Research and Development Center of Hebei Universities Langfang Hebei People's Republic of China; ^4^ College of Light Industry and Food Engineering Nanjing Forestry University Nanjing People's Republic of China

**Keywords:** antioxidant, edible fungi, foxtail millet, phenolic compounds, physical and functional properties, solid‐state fermentation

## Abstract

Foxtail millet is typically dehulled before consumption or processing. However, foxtail millet bran also contains abundant phenolic compounds and other nutrients. Edible fungi have rich extracellular enzyme systems; are environmentally friendly and safe for consumption; and have been shown to effectively degrade lignin and cellulose. This study aimed to screen edible fungi that can effectively ferment undehusked foxtail millet, improving its nutritional value and antioxidant properties through solid‐state fermentation (SSF). The results demonstrated that fermentation utilizing *Pleurotus geesteranus* exhibited significant improvements in both the phenolic compound content and antioxidant properties of foxtail millet, with the optimal fermentation period determined to be 30 days. The physical and functional properties of fermented undehusked foxtail millet (FFM) flour were effectively improved, increasing crude protein, vitamin C, and crude polysaccharide contents by 11.46%, 27.78%, and 54.17%, respectively. In vitro scavenging activities of FFM were 73.19%, 93.86%, and 63.75% for 2,2‐diphenyl‐1‐picrylhydrazyl (DPPH), 2,2′‐azino‐bis(3‐ethylbenzothiazoline‐6‐sulfonic acid) (ABTS·^+^), and superoxide anion radicals (O_2_ ^−^), respectively. The total antioxidant capability (T‐AOC) and superoxide dismutase (SOD) activity of FFM were 1.01 mM Trolox equivalents (TE)/g and 89.05 U/g, respectively. Additionally, T‐AOC, SOD, and glutathione peroxidase (GSH‐Px) activities increased, whereas malondialdehyde (MDA) levels decreased in the heart, liver, and kidneys of mice treated with FFM flour, indicating enhanced antioxidant capacity. Therefore, fermentation with edible fungi is suitable for improving the nutritional composition and antioxidant properties of foxtail millet.

## Graphical abstract

Fermentation of undehusked foxtail millets with *P. geesteranus* significantly increases the content of phenolic compounds and enhances its antioxidant properties. The physical and functional characteristics, as well as the nutritional value, of the fermented foxtail millets are improved.

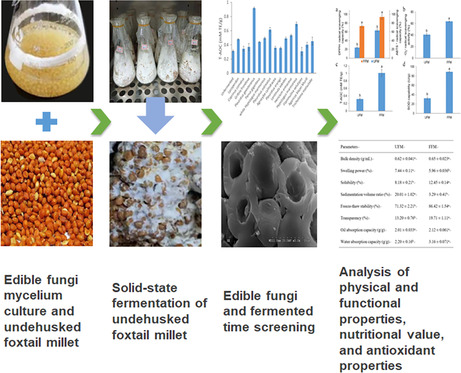

## 1 INTRODUCTION

Millets serve as a significant food source for people in arid regions worldwide and are considered one of the most crucial drought‐resistant crops, ranking sixth among cereal crops (Amadou et al., 2014). Foxtail millet, commonly referred to as Chinese millet, Italian millet, or German millet, is a prevailing cultivar of millet extensively grown across various regions of Asia, particularly India and China, as well as in Europe (Nazni & Shobana, 2016). It plays a pivotal role in agriculture and food security in numerous developing countries due to its unique ability to thrive in regions characterized by extreme heat and limited rainfall. China, having cultivated foxtail millet for over 6000 years, stands as the world's third‐largest producer after India and Nigeria (Amadou et al., 2014).

Foxtail millet is abundant in high‐quality protein, dietary fiber, and various vitamins (such as B‐complex vitamins and vitamins A and E). It is also rich in phenolic acids, carotenoids, tannins, flavonoids, and other components, making it a natural source of antioxidants (Xiang et al., 2019a). Recognized for its high digestibility and low allergenicity, foxtail millet is typically subjected to milling to remove the husk. The resulting millet grains can then be cooked and prepared as porridge, or ground into flour for use in specialty foods (such as snacks and alcoholic beverages) (Amadou et al., 2011a, 2014). However, the husks are abundant in phenolic compounds and are a reservoir of natural antioxidants and α‐glucosidase inhibitors with potential health benefits (Zhang et al., 2021). Accounting for 13.5% of the millet's composition, the husk holds remarkable value, and fully utilizing its phenolic substances is essential for enhancing the value of foxtail millet. The husk and aleurone layers of foxtail millets serve are the primary repositories for phenolic compounds (Chandrasekara et al., 2012).

Fermentation can induce physical and chemical changes in products and has been demonstrated to effectively increase the nutritional content and reduce anti‐nutritional factors of millet (Amadou et al., 2014). In particular, solid‐state fermentation (SSF) offers distinct advantages, including low cost, simple operation, and environmental friendliness. The mycelia of edible fungi can produce various enzymes (such as cellulase, laccase, lipase, and amylase), which reduce the lignin and crude fiber contents in the culture medium and release bound phenolics, thereby increasing the overall free phenolic content and enhancing the antioxidant activity. SSF with edible fungi has been beneficial in enhancing nutrition and antioxidant capacity of various cereals, as well as facilitating the release of phenolic compounds (Acosta‐Estrada et al., 2019). For example, the addition of *Ganoderma sinense* effectively increases the levels of dietary fiber, lysine, and protein of corn (Lou et al., 2023), while lentils fermented with *Pleurotus ostreatus* exhibit increased antioxidant activity and proteolysis (Asensio‐Grau et al., 2020). Moreover, *Flammulina velutipes, P. ostreatus*, and *Hericium erinaceus* have been used to enhance the antioxidant capacity, protein profile, and processing potential of soybean meal (Wang et al., 2023). Fermentation with *P. ostreatus* is highly effective in promoting the release of polyphenols, enhancing antioxidant activity and protein content, and reducing the phytate content in white quinoa and pardina lentils (Sánchez‐García et al., 2022). Edible fungi are recognized as valuable sources of nutrition and can thrive on lignocellulosic substrates, making them well suited for the degradation of various substrates, such as grains, legumes, and seeds. This fermentation approach offers a promising method for enhancing the nutritional quality and functional properties of these food ingredients.

Therefore, we hypothesized that SSF fermentation with edible fungi will enhance nutrition, increase free phenolic content, and improve antioxidant capacity of the fermented undehusked foxtail millet (FFM). This study aimed to screen edible fungi that can effectively ferment undehusked foxtail millet and improve its nutritional value and antioxidant properties through SSF.

## 2 MATERIALS AND METHODS

### 2.1 Microorganisms

Fifteen strains of edible fungi (*Coprinus comatus, Lycoperdon* spp., *Grifola frondosa, Pleurotus geesteranus, Panus giganteus, P. ostreatus, Agrocybe cylindracea, Pleurotus eryngii, Lentinula edodes, H. erinaceus, Hypsizygus marmoreus, F. velutipes, Agaricus blazei Murill*, and *Tricholoma matsutake*) were obtained from the Edible and Medicinal Fungi Research and Development Center of Hebei University, China. All the edible fungi mycelia were cultured on potato dextrose agar medium at 25°C.

### 2.2 Materials and chemicals

Foxtail millet (Huangqi millet) without husking was purchased from Fengning County (Chengde, China). All necessary chemicals and reagents were procured from Sinopharm Chemical Reagent Co. Ltd. (Shanghai, China). Deionized water was used for all experimental procedures.

### 2.3 SSF and treatment of foxtail millet

When the mycelia were full of potato dextrose agar plate, a liquid‐state fermentation was initiated by adding liquid potato dextrose broth (100 mL). The mycelia were then cultured (25°C) and shaken (150 rpm) for 7 days.

Foxtail millet was washed and soaked in water (25°C) for 18 h and excess water was removed from the surface. It was then sterilized (121°C for 30 min), inoculated with 10% (w/v) homogeneous mycelial suspension, and incubated (25°C) for 5–80 days. Control samples were treated under identical conditions, with sterile deionized water used, instead of mycelia (unfermented undehusked foxtail millet: UFM).

The millets were heat‐dried at 50°C until a stable weight was achieved, then ground into powder, and stored at −20°C until use.

### 2.4 Extraction and determination of phenolics

Phenolic compounds were extracted followed a previously described method, with modifications (Sharma et al., 2018). Millet powder was extracted with 65% ethanol (1:20, m/v) under ultrasonication (60 min) to obtain free phenolic compounds. The residue was treated with sodium hydroxide (NaOH) (2 M) at 25°C for 1 h. To obtain the bound phenolics, pH of the mixture was adjusted to 2.5 by adding HCl, and the resulting solution was subjected to three consecutive extractions, using ethyl acetate (10 mL) each time.

The total phenolic content was determined using Folin–Ciocalteu reagent. The absorbance of samples was measured (760 nm) using a UV‐L4 spectrophotometer (Yoke Instrument Co., Ltd., Shanghai, China). The phenolic content of each fraction was quantified by employing the gallic acid standard curve. The results were presented as milligrams (mg) of gallic acid equivalents (GAEs) per 100 g of sample (mg GAE/100 g).

### 2.5 In vitro antioxidant activity of phenolic extracts in FFM

#### 2.5.1 Assay for 2,2‐diphenyl‐1‐picrylhydrazyl (*DPPH·*), 2,2′‐azino‐bis(3‐ethylbenzothiazoline‐6‐sulfonic acid) (*ABTS·* ^*+*^), and superoxide anion radical (O_2_ ^−^) free scavenging radical activity

The extract of phenolic compounds was used for subsequent determination of free radical scavenging ability. The DPPH solution was added to the sample (1:6, v/v), mixed, and stored in the dark for 30 min (Sharma et al., 2021). The sample (0.1 mL) was then added to the ABTS working solution (1.9 mL) and stored in the dark for 5 min. The sample (1 mL) was added to 0.05 M Tris–HCl (4.5 mL) along with 2.5 mM pyrogallol (0.4 mL), then it was mixed and reacted for 5 min (Amadou et al., 2011b). The absorbances of the samples were measured at 517, 734, and 299 nm, respectively, and calculated as follows:

(1) $$\begin{array}{c} \text{scavenging activity}\;\left(\%\right)=\\ \;\frac{\text{Absorbance of blank}-\text{Absorbance of sample}}{\text{Absorbance of blank}}\times 100 \end{array}$$

#### 2.5.2 Superoxide dismutase (SOD) and total antioxidant capability (T‐AOC) assay

A SOD assay kit and an antioxidant capacity assay kit were used to measure the SOD activity and T‐AOC (Nanjing Jiancheng Bioengineering Institute, Nanjing, China). The absorbances of the samples were recorded at 450 and 520 nm, respectively.

### 2.6 Scanning electron microscopy (SEM) of FFM

Undehusked foxtail millet fermented with mycelia and unfermented control samples were analyzed using SEM (S‐4800 High Resolution SEM, Hitachi, Japan) at an acceleration voltage of 20 kV. Before observation, the millets were subjected to heat‐drying, until a consistent weight was achieved. Then, they were cut in half and coated with gold (Au) before SEM imaging (Zheng et al., 2022).

### 2.7 Physical and functional properties of FFM

#### 2.7.1 Bulk density assay

A graduated cylinder was filled with millet powder up to the 10 mL mark. The weights of the cylinder and powder were recorded to calculate the bulk density (g/mL).

#### 2.7.2 Freeze–thaw stability assay

The millet powder was mixed with water to prepare an emulsion (6%, m/v), which was heated at 100°C for 20 min, and then cooled to 25°C. The resulting paste (m_1_) was weighed, transferred into a centrifuge tube, and frozen at −18°C for 24 h. Subsequently, the paste was thawed for 3–5 h at 25°C and subjected to centrifugation (3000 × *g*) for 20 min. The supernatant was then removed to accurately weigh the precipitate (referred to as ‘m_2_’). The rate of water evolution was calculated as follows (Lin et al., 2019):

(2) $$\text{Rate of water output}\;\left(\%\right)=\frac{m_{1}-m_{2}}{m_{1}}\times 100$$

#### 2.7.3 Transparency and settleability assay

The millet powder was mixed with water to prepare an emulsion (1%, m/v), which was heated at 100°C for 30 min, and then cooled to 25°C. The light transmittance was measured (620 nm) using water as a blank (Lin et al., 2019).

The paste was transferred into a calibration tube to record the initial volume. It was then kept at 25°C for 24 h to allow for sedimentation, and the volume of sedimentation was measured and recorded. The settleability was expressed as the sedimentation volume ratio, which is the ratio of volume of the sedimentation to the initial total paste volume (Lin et al., 2019).

#### 2.7.4 Swelling and solubility assay

Water was added to millet powder to create a suspension (2%, m/v) and kept in a water bath (95°C, 30 min) with constant stirring. Subsequently, the suspension was cooled to 25°C, subjected to centrifugation (3000 × *g*) for 20 min, transferred to a Petri dish with a known weight, and dried at 105°C, until a constant weight was reached. The weights of the dried supernatant and sediment were recorded. The calculation was performed as follows:

(3) $$\text{Solubility}\;\left(\%\right)=\frac{\mathrm{Dry}\;\text{supernatant weight}}{\text{Weight of millet powder}}\times 100$$

(4) $$\text{Swelling power}\;\left(\%\right)=\frac{\text{Sediment weight}}{\mathrm{Dry}\;\text{sample weight}\times \left(100-\text{Solubility}\%\right)}\times 100$$

#### 2.7.5 Oil and water absorption capacity assay

Water was added to the millet powder (10%, w/v) in a centrifuge tube of known weight, kept aside at 25°C for 30 min, and centrifuged (3000 × *g*) for 20 min. The supernatant was then removed. The water absorption capacity of millet powder was determined by measuring the difference between the initial mass and final mass after removing water. For the oil absorption capacity, soybean oil was substituted for water in the aforementioned procedure.

### 2.8 Nutritional value of FFM

#### 2.8.1 Crude protein, crude fat, crude fiber, and crude polysaccharide assays

Crude protein content was measured and calculated as previously described (González Martín et al., 2014) with a conversion factor of 6.25. A Soxhlet apparatus and petroleum ether were used to extract the crude fat, with the solvent subsequently removed using a rotary vacuum evaporator at 50°C. The estimation of crude fiber in millet was performed through acid/alkaline hydrolysis of insoluble residues (González Martín et al., 2014). The rapid enzymatic assay method (Asp et al., 1983) was used to determine the total dietary fiber. Soluble fiber was calculated by subtracting crude fiber from the total dietary fiber. The phenol–sulfuric acid method (Zhu et al., 2012) was used to determine the crude polysaccharide content.

#### 2.8.2 Vitamin C and phytic acid assays

Vitamin C was extracted from the millet powder using metaphosphoric acid solution (2%, w/v) and quantified by titrating against a 2,6‐dichloroindophenol solution.

The phytic acid content in millet powder was measured following a method described by Shi et al. (2018). The absorbance of the sample was measured at 500 nm, calculated using a phytic acid standard curve.

### 2.9 Animals and experimental design

All experimental protocols were approved by the Animal Research Committee of the Langfang Normal University (Langfang, China). Forty ICR mice weighing 30–35 g and aged 8 weeks were randomly allocated to one of the four groups (10 mice each) and housed in a dedicated room where the temperature was maintained within a range of 21–25°C under a 12:12 light and dark cycle. The naïve group, named AC, received a regular chow diet. The positive control group, named PC, was given a diet comprising a mixture of regular chow and vitamin C (50 mg/kg body weight). The two test groups were given a diet composed of a mixture of regular chow and UFM (the UFM group) or a mixture of regular chow and FFM (the FFM group), each with a millet content of 200 g/kg. All mice and diets used in this study were prepared by SPE Biotechnology Co., Ltd (Beijing, China). The mice were fed their respective diets for 30 days with body weights recorded on a weekly basis and daily intake of feed carefully recorded.

### 2.10 Preparation of tissues

All mice were sacrificed by decapitation at the end of the experiment. Their tissues (livers, hearts, and kidneys) were immediately frozen using liquid nitrogen and stored at −80°C until further analysis. The tissue sample was homogenized using precooled saline solution at a ratio of 1 mL per 100 mg of tissue and centrifuged (3000 × *g*) at 4°C for 15 min. The supernatants were carefully collected and stored at 4°C for subsequent analyses.

### 2.11 Antioxidant activity measurements in different tissues

The supernatants were used for in vitro antioxidant activity analysis. Commercial assay kits from Nanjing Jiancheng Bioengineering Institute were employed to assess the levels of malondialdehyde (MDA), SOD, T‐AOC, and glutathione peroxidase (GSH‐Px) activity. MDA levels were quantified and expressed as nmol/mg protein. SOD content, T‐AOC, and GSH‐Px activity were determined and expressed as U/mg protein.

### 2.12 Enzyme activity assay

Foxtail millet samples coated with mycelium at different fermentation times were homogenized with 0.9% NaCl (1:2, w/v) at 4°C and centrifuged (3000 × *g*) for 10 min. The supernatant of the sample was used for enzyme assays. The mycelia, which were shaken at 150 rpm (revolutions per minute) for 7 days, were used as the control (0 day).

The activity of laccase was assessed by monitoring the absorbance change at 420 nm, which is directly associated with the rate of ABTS oxidation (Songulashvili et al., 2007). Cellulase, hemicellulose, and amylase activities were determined using the 3,5‐dinitrosalicylic acid assay (Doria et al., 2014). A spectrophotometric method (410 nm) was used to measure polyphenol oxidase (PPO) activity (Lin et al., 2022).

Enzyme activity results were expressed as U/g, where U represents the amount of enzyme capable of catalyzing the conversion of 1 mol of substrate per minute.

### 2.13 Statistical analysis

All tests were conducted in triplicate, and the obtained data were presented as means ± standard deviation, providing information about the central tendency and variability of the measurements. Statistical analyses were conducted using SPSS Statistics 23 software. Statistical significance was assessed using analysis of variance (ANOVA), with a significance level of *p* < .05 was considered significant.

## 3 RESULTS AND DISCUSSION

### 3.1 Screening of edible fungi for increasing antioxidant activity of undehusked foxtail millet by SSF

**FIGURE 1:**
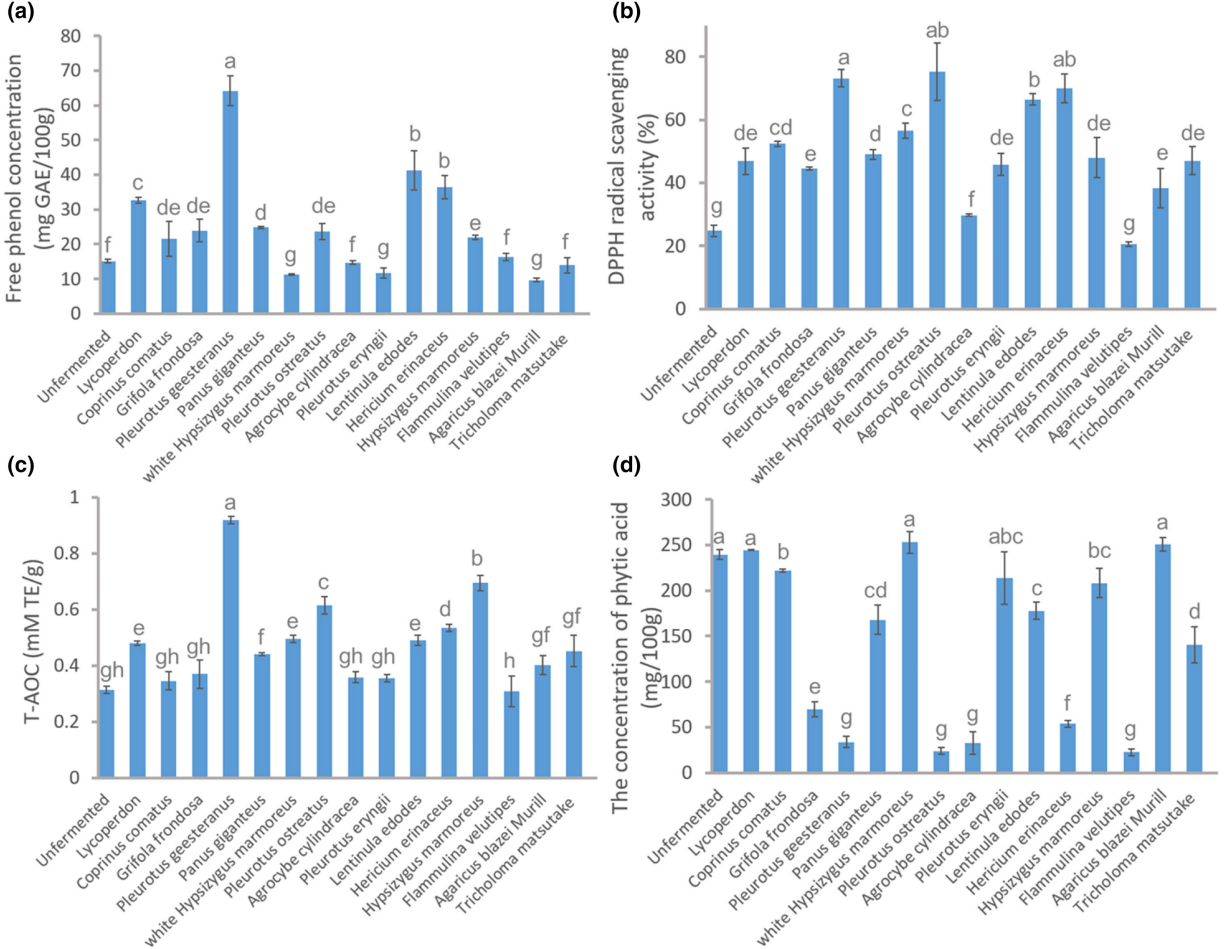
Screening antioxidant activity in undehusked foxtail millet fermented with 15 types of edible fungi. (a) Free phenol concentration. (b) 2,2‐Diphenyl‐1‐picrylhydrazyl (DPPH) radical scavenging activity. (c) Total antioxidant capability (T‐AOC). (d) Phytic acid concentration. Different letters on the bar charts represent significant differences (*p* < .05). GAE, gallic acid equivalent.

Phenolic compounds play a crucial role as primary antioxidants, directly contributing to the overall antioxidant capacity of foxtail millet, which can be enhanced through fermentation processing (Balli et al., 2020). Therefore, we screened for edible fungi that could increase the phenolic compounds content in undehusked foxtail millet through SSF. As shown in Figure 1a, after SSF with *C. comatus, Lycoperdon* spp., *G. frondosa, P. geesteranus, P. giganteus, P. ostreatus, L. edodes, H. erinaceus*, and *H. marmoreus*, the free phenol content in foxtail millet increased significantly compared to that in UFM, which was only 15.10 ± 0.54 mg GAE/100 g. Among these treatments, *P. geesteranus*–FFM had the highest free phenol content (64.22 ± 4.33 mg GAE/100 g). These findings demonstrate that the SSF of foxtail millet with edible fungi can increase the content of free phenols, and *P. geesteranus* may have an advantage in enhancing the production of phenolic compounds during SSF.

Foxtail millets's health benefits primarily stem from its antioxidant properties (Xiang et al., 2019b). To distinguish the antioxidant properties of FFM with different edible fungi, we determined the DPPH radical scavenging activity and T‐AOC (Figure 1b,c). The DPPH radical scavenging activities of foxtail millet were significantly increased following fermentation by all the different edible fungi tested, except for *F. velutipes*. Foxtail millets fermented by *P. geesteranus*, *P. ostreatus*, *H. erinaceus*, and *L. edodes* exhibited the highest DPPH scavenging activity (73.19 ± 2.79%, 75.21 ± 9.08%, 69.92 ± 4.52%, and 66.47 ± 1.77%, respectively), while that of UFM was only 24.79 ± 1.89%. As shown in Figure 1c, T‐AOC of FFM fermented by *P. geesteranus*, *Lycoperdon* spp., *P. giganteus*, *H. marmoreus*, *P. ostreatus*, *L. edodes*, *H. erinaceus*, and *H. marmoreus* increased significantly compared to that of UFM. Among these, *P. geesteranus* exhibited the highest T‐AOC (0.92 ± 0.013 mM Trolox equivalents [TE]/g), 2.97 times that of UFM (0.31 ± 0.012 mM TE/g). This result indicates that *P. geesteranus* is a promising candidate for enhancing FFM's antioxidant properties.

In addition to its antioxidant properties, the rich nutritional content of millet is an important contributor to its health benefits. However, foxtail millet also contains many anti‐nutrients (such as phytic acid), which can combine with nutrients to inhibit their absorption in the husk and aleurone layers. Fermentation is an effective approach for reducing the phytic acid content and enhancing the nutritional value of millets (Azeez et al., 2022). To determine the optimal edible fungi for fermenting foxtail millet, the phytic acid content was quantified in FFM fermented by the different edible fungi (Figure 1d). The phytic acid content in FFM fermented by *Lycoperdon*, *H. marmoreus*, *Pleurotus eryngii*, and *A. blazei* (approximately 245 mg/100 g) was not significantly different compared to that in UFM (239.55 ± 5.31 mg/100 g). However, the phytic acid contents in FFM fermented by *P. geesteranus*, *P. ostreatus*, *Agrocybe cylindracea*, and *F. velutipes* were reduced, at 33.68 ± 6.43 mg/100 g, 23.93 ± 4.05 mg/100 g, 32.32 ± 12.22 mg/100 g, and 22.31 ± 3.86 mg/100 g, respectively, indicating that these four edible fungi can effectively enhance the nutritional value of millet.

Based on these experimental results, *P. geesteranus* showed the strongest potential for enhancing antioxidant and nutritional properties in FFM through SSF and was selected in subsequent experiments.

### 3.2 Effect of fermentation time on phenolic compound content of foxtail millet

**FIGURE 2:**
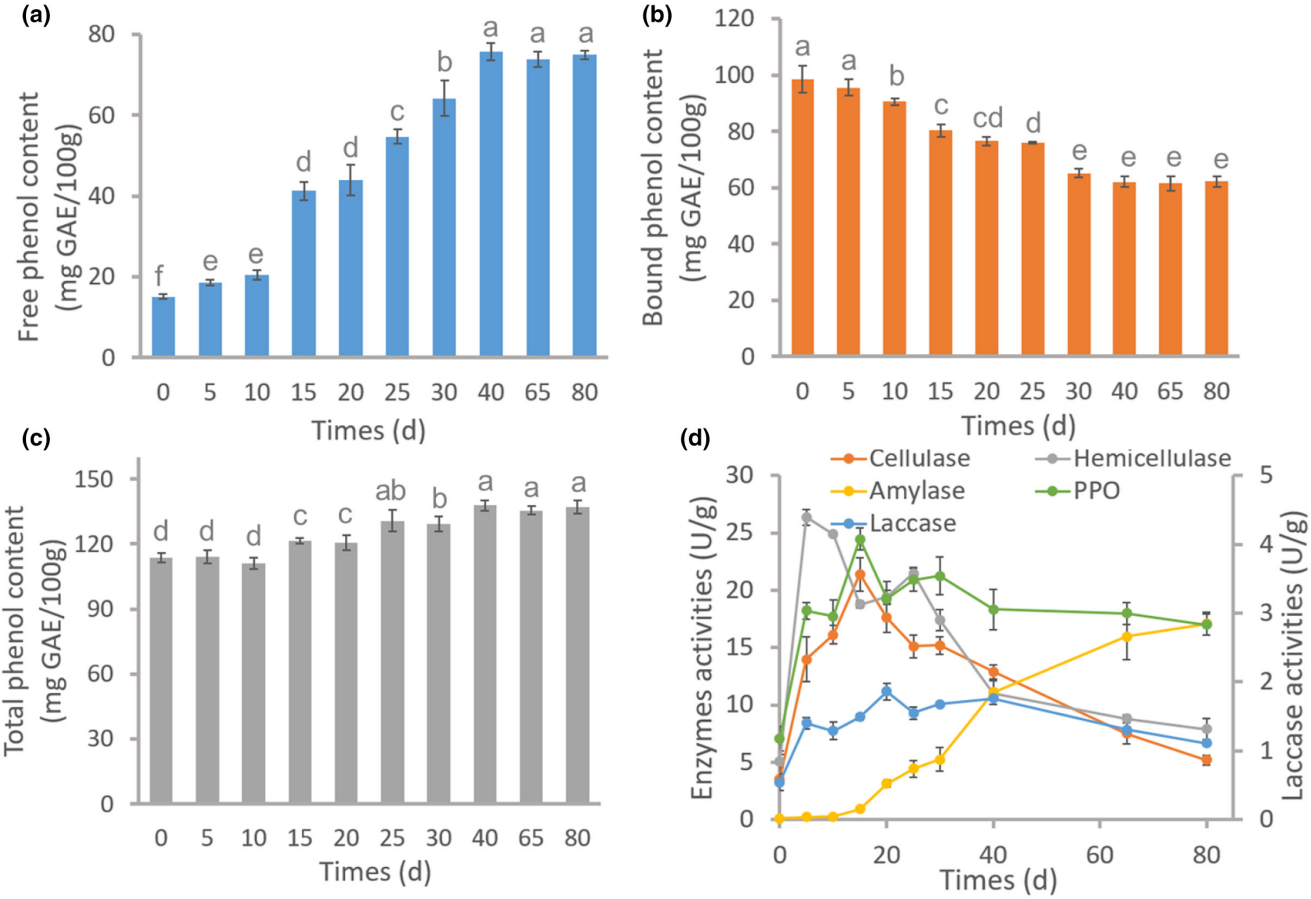
Phenolic compound content of foxtail millet and *Pleurotus geesteranus* enzyme activity following different fermentation times. (a) Free phenol content. (b) Bound phenol content. (c) Total phenol content. (d) *P. geesteranus* enzyme activity. 0 day represents the enzyme activity of mycelia, which were cultured at 25°C and shaken at 150 rpm for 7 days. Different letters on the bar charts represent significant differences (*p* < .05).

The duration of fermentation significantly influenced the content of phenolic compounds in FFM (Figure 2). As fermentation time increased, the content of free phenol compound in FFM fermented by *P. geesteranus* gradually increased (Figure 2a). When the fermentation time reached 40 days, the millet had its highest free phenol content (75.79 ± 2.21 mg GAE/100 g), over four times higher than that of the UFM (15.10 ± 0.54 mg GAE/100 g). Subsequently, free phenol content in millet did not significantly change with prolonged fermentation time, possibly due to decreased nutrient availability or prerequisite enzyme activity during prolonged fermentation (Balli et al., 2020; Lou et al., 2023).

Contrary to the trend in free phenol content, bound phenol content in FFM fermented with *P. geesteranus* gradually decreased with increasing fermentation time (Figure 2b). At 30 days of fermentation, foxtail millet had the lowest bound phenol content (65.05 ± 4.33 mg GAE/100 g) compared to that of the control sample (98.59 ± 4.72 mg GAE/100 g). Thereafter, bound phenol content remained relatively stable there with extended fermentation times (*p* > .05). Most bound phenols in grain bran are associated with hemicellulose, cellulose, or polysaccharides present in cell walls (Acosta‐Estrada et al., 2019). During the fermentation process, edible fungi generate a diverse range of enzymes, including cellulase, hemicellulase, and laccase. These enzymes have the ability to degrade the cell walls of grains by destroying ester bond linkages between hemicellulose, cellulose, or polysaccharides with phenolic acid carboxylic groups, facilitating the release of bound phenolic substances (Acosta‐Estrada et al., 2019). This explains why the content of bound phenols in this study decreased with increasing fermentation time. However, after 65 days and 80 days of fermentation, the bound phenol content in FFM showed no significant changes, possibly due to decreased activity of extracellular enzymes secreted by *P. geesteranus* mycelium at this time.

Figure 2c illustrates the variation in total phenolic compounds content in FFM during different fermentation durations. Notably, there is no significant alteration in the total phenolic compound content in the first 10 days of fermentation. However, the total phenolic content gradually increased between 10 days and 40 days of fermentation, reaching a peak of approximately 140 mg GAE/100 g between days 25 and 40. Subsequently, total phenolic compound content remains nearly constant with the extension of fermentation time beyond this peak value. The increase in total phenolic content observed in FFM can be attributed to the metabolic activity of edible fungi. Edible fungi utilize soluble and fermentable fibers for growth, releasing phenolic compounds that are “mechanically trapped” within the polymer structure of the fibers, resulting in an increased phenolic content in FFM (Balli et al., 2020). Changes in soluble fibers in foxtail millet before and after fermentation were detected, with soluble fiber content decreasing by 94.89% after fermentation (Figure S1). These results demonstrate that fermentation is a useful processing method to enhance the content of phenolic compounds in millets.

The activities of cellulase, hemicellulase, amylase, laccase, and PPO were measured during fermentation. As shown in Figure 2d, except for amylase, the activities of the other enzymes gradually decreased after 40 days of fermentation, potentially contributing to the lack of significant changes in phenolic compound content after 40 days of fermentation. Cellulase, hemicellulase, PPO, and laccase play important roles in degrading cellulose and lignin in foxtail millet husks, and the activities of these enzymes are optimal at 15 and 30 days of fermentation. The activity of amylase is optimal during the later stages of fermentation, which may be attributed to the depletion of nutrients such as cellulose and lignin, leading to the utilization of starch by edible fungi for growth. Based on variations in the phenolic compound content of FFM and enzyme activities of mycelia at different fermentation times, the optimal fermentation time may be between 30 and 40 days. During this fermentation period, the content of phenolic substances was relatively high, and the enzyme activity was optimal.

### 3.3 SEM images of foxtail millet throughout fermentation

**FIGURE 3:**
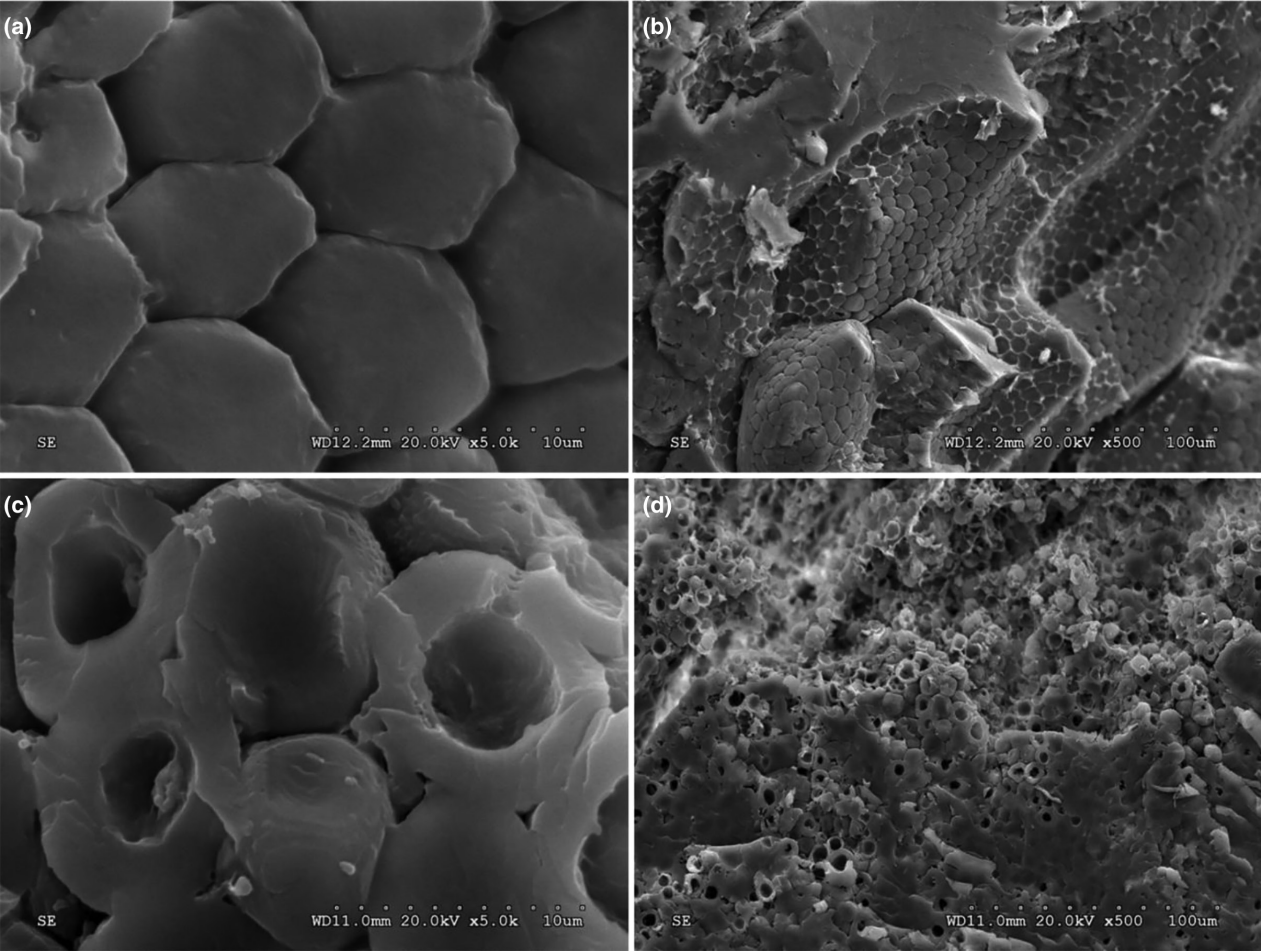
Scanning electron microscopy (SEM) images of fermented foxtail millet's internal structure (a) after 30 days of fermentation at 20 kV extra high tension (EHT), 12.2 mm work distance (WD), and 10 μm size; (b) after 30 days of fermentation at 20 kV EHT, 12.2 mm WD, and 100 μm size; (c) after 40 days of fermentation at 20 kV EHT, 12.2 mm WD, and 10 μm size; and (d) after 40 days of fermentation at 20 kV EHT, 12.2 mm WD, and 100 μm size.

Scanning electron microscopy (SEM) has provided valuable and effective insights into the microstructures of grains during fermentation. Figure S2 and Figure 3 depict the internal morphological properties of FFM throughout fermentation. The results revealed that intact starch granules were densely embedded in the matrix within 30 days of fermentation (Figure 3a,b, and Figure S2), with no significant difference from the control sample (Figure S2). However, at 40 days of fermentation, degradation of internal starch granules in the FFM led to the formation of surface “pores” (Figure 3c,d), possibly due to amylase action. With prolonged fermentation time (65 and 80 days), starch granules’ defects worsened, indicating severe degradation by *P. geesteranus*. This finding aligns with the observed increase in amylase activity during the later phases of fermentation.

Starch, comprising 60%–70% of total grain weight, is the primary carbohydrate in millet and determines the quality of millet‐based products (Ingle et al., 2023). To minimize the impact of SSF on the quality of foxtail millet, it is necessary to shorten the fermentation time. Based on the changes in phenolic compounds (Figure 2) and internal morphology (Figure 3) of the FFM, the optimal fermentation time for *P. geesteranus* SSF of foxtail millet was determined to be 30 days.

### 3.4 Physical and functional properties of FFM

When millet is used to produce extrusion‐cooked and baked products, it is often present in flour form. Therefore, researching the physical and functional changes in FFM flour is of great significance. As shown in Table 1, the bulk density of FFM and UFM ranged from 0.65 ± 0.023 g/mL to 0.62 ± 0.041 g/mL, respectively, with no significant difference (*p* > .05), indicating that fermentation by *P. geesteranus* did not affect the particle size and density of foxtail millet flour. Settlement is an intrinsic factor influencing flour paste and is typically measured by the sedimentation volume ratio. The sedimentation volume ratio of the FFM paste was 1.21 times that of the UFM paste (Table 1), indicating that the FFM paste had a higher stability. This increase may be associated with the relative content of amylose and amylopectin as well as the size and length of the molecular chain of resistant starch (Wang et al., 2022). The increased sedimentation volume ratio of the FFM may be due to the disruption of the molecular bonding of starch, leading to the conversion into molecular chains of suitable lengths and increasing the degree of intermolecular aggregation during fermentation (You et al., 2019). Assessing freeze–thaw stability is crucial in ensuring the quality of starch‐based frozen convenience foods, as it determines the flour's capability to endure potential unfavorable physical alterations during the freeze–thaw process. The freeze–thaw stability of UFM (71.32 ± 2.21%) was lower than that of FFM (86.42 ± 1.54%), indicating better stability in UFM. This finding may be associated with the increased amylose content in foxtail millet due to fermentation, as there is a negative correlation between amylose content and freeze–thaw stability (Yang et al., 2019). Transparency, related to aging and usually expressed as transmittance, is an important quality factor in food processing. As shown in Table 1, FFM showed higher transparency (19.71%) than UFM (13.20%), possibly due to variations in the amylose content of the flour, which reduces translucency of the starch paste (Yang et al., 2019).

**TABLE 1:** Effects of fermentation with *Pleurotus geesteranus* on physical and functional properties of undehusked foxtail millet.

Parameters	UFM	FFM
Physical and functional properties		
Bulk density (g/mL)	0.62 ± 0.041^a^	0.65 ± 0.023^a^
Swelling power (%)	7.44 ± 0.11^a^	5.96 ± 0.036^b^
Solubility (%)	8.18 ± 0.21^b^	12.45 ± 0.14^a^
Sedimentation volume ratio (%)	20.01 ± 1.02^a^	3.29 ± 0.41^b^
Freeze–thaw stability (%)	71.32 ± 2.21^b^	86.42 ± 1.54^a^
Transparency (%)	13.20 ± 0.76^b^	19.71 ± 1.11^a^
Oil absorption capacity (g/g)	2.01 ± 0.033^a^	2.12 ± 0.061^a^
Water absorption capacity (g/g)	2.20 ± 0.16^b^	3.16 ± 0.071^a^
Nutritional value		
Crude protein (g/100 g)	10.21 ± 0.073^b^	11.38 ± 0.16^a^
Crude fiber (g/100 g)	6.98 ± 0.15^a^	5.07 ± 0.046^b^
Phytic acid (mg/g)	2.40 ± 0.051^a^	0.12 ± 0.020^b^
Vitamin C (mg/100 g)	0.72 ± 0.032^b^	0.92 ± 0.028^a^
Crude fat (g/100 g)	3.14 ± 0.024^a^	1.93 ± 0.016^b^
Crude polysaccharide (mg/100 g)	0.24 ± 0.0021^b^	0.37 ± 0.0045^a^

*Note*: Values represent the means ± standard deviation (SD) of three replicates. Means in a row followed by different superscript letters indicate significant differences (*p* < .05).

Abbreviations: FFM, undehusked foxtail millet fermented by *P. geesteranus*; UFM, unfermented undehusked foxtail millet.

Water absorption capacity refers to the ability of flour to absorb a certain quantity of water to achieve the optimal result or desired consistency. The water absorption capacity of FFM increased by 43.64% after fermentation (Table 1). This increase might be attributed to the improved protein value/quality (Azeez et al., 2022), along with the alterations in the starch granule surface structure resulting from fermentation in the flour (Lin et al., 2019). This enhancement improves water utilization efficiency, which plays a significant role in starch gelatinization, affecting the final texture of cooked products (Rao et al., 2021). Swelling power and solubility significantly influence the processing characteristics of starch‐based foods. Researchers have discovered that there is an inverse relationship between swelling power and moisture content (Li et al., 2019; Rao et al., 2021). The UFM has a larger swelling power than FFM (Table 1), possibly because fermentation alters the surface structure of the starch granules, allowing them to adsorb more water, thereby reducing the swelling power. FFM flour solubility (12.45 ± 0.14%) is larger than that of UFM (8.18 ± 0.21%), attributed to the degree of structural weakening and depolymerization of starch granules due to fermentation (Rao et al., 2021). The oil absorption capacity of flour is essential for preserving flavor and enhancing mouthfeel. However, there were no notable distinctions in the oil absorption capacity observed between FFM and UFM, indicating that fermentation did not compromise the flavor or texture of foxtail millet‐based products.

### 3.5 Nutritional composition of FFM

Nutritional composition analysis revealed significant differences between FFM and UFM (Table 1). After fermentation, crude protein, vitamin C, and crude polysaccharide contents were significantly increased by 11.46%, 27.78%, and 54.17%, respectively, compared with that of UFM. The crude protein content of FFM was 11.38 ± 0.16 g/100 g, higher than that of the UFM (10.21 ± 0.073 g/100 g); this enhancement may be attributed to the synthesis of proteolytic enzymes produced by edible fungi during SSF (Mudau et al., 2022; Nkhata et al., 2018). Vitamin C content increased from 0.72 mg/100 g to 0.92 mg/100 g after fermentation (Table 1). Since glucose is a precursor of vitamin C formation (Nkhata et al., 2018), the elevated vitamin C content in millets during the fermentation process may be attributed to the enzymatic hydrolysis of starch by diastases and amylases. This enzymatic activity leads to an increased availability of glucose, thereby enhancing vitamin C biosynthesis (Nkhata et al., 2018). In addition to crude protein and vitamin C, the crude polysaccharide content in the FFM significantly enhanced from 0.24 mg/100 g to 0.37 mg/100 g. This may be attributed to the degradation of substances in the millet bran, such as cellulose, bound phenols, and lignin, by extracellular enzymes secreted by edible fungi.

In contrast, the levels of crude fiber, phytic acid, and crude fat in millet were reduced after fermentation (Table 1). Crude fat and crude fiber contents of FFM decreased by 38.54% and 27.36%, respectively, consistent with previous findings (Mudau et al., 2022). The observed decrease in fat content in the FFM may be attributed to two potential factors: the oxidation of fat by edible fungi to generate energy for their activities, and lipolytic hydrolysis facilitated by lipases during the fermentation process (Adebiyi et al., 2019). The crude fiber content exhibited a significant reduction (*p* < .05) from 6.98 ± 0.15 g/100 g in the UFM to 5.07 ± 0.046 g/100 g in the FFM. This decline may be attributed to the extracellular enzymes secreted by *P. geesteranus* during the SSF process, which degrade the fiber components as a carbon source for their own growth. The greatest decrease observed in the FFM was in phytic acid concentration (95%), which could be attributed to the action of phytase, as it hydrolyzes phytic acid into lower inositol phosphates. Phytic acid is typically found in grains, with a higher concentration in bran. Excessive phytic acid can negatively impact mineral absorption and is generally considered an anti‐nutritional factor. Therefore, the considerable reduction in phytic acid content in FFM indicates the potential of SSF by edible fungi to enhance the nutritional value of foxtail millet.

### 3.6 Antioxidant capability of FFM in vitro

**FIGURE 4:**
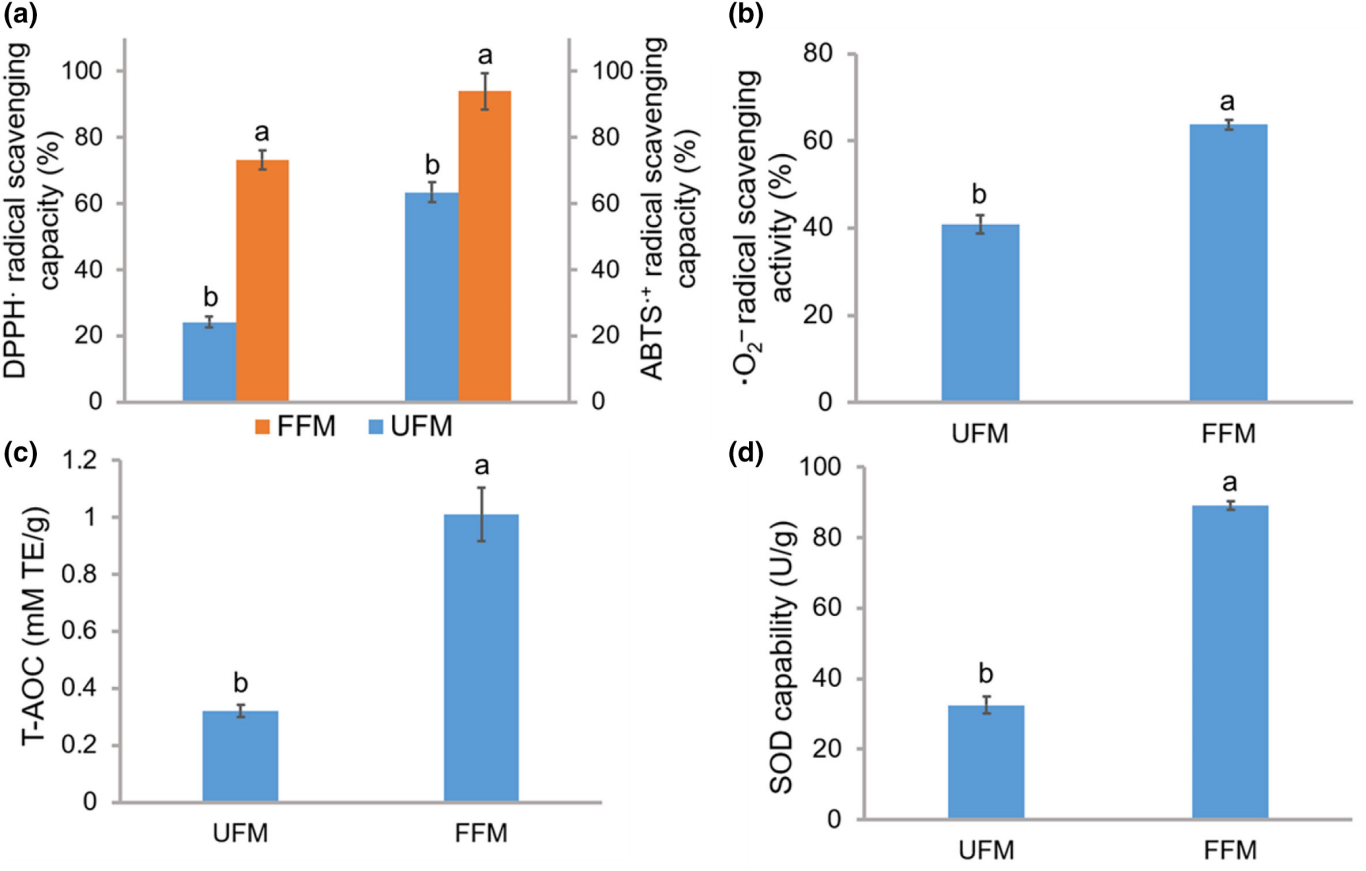
Antioxidant activity of fermented foxtail millet in vitro. (a) 2,2‐diphenyl‐1‐picrylhydrazyl (DPPH·) and 2,2′‐azino‐bis(3‐ethylbenzothiazoline‐6‐sulfonic acid (ABTS·^+^) radical scavenging capacities. (b) ·O_2_^−^ scavenging activity. (c) Total antioxidant capability (T‐AOC). (d) Superoxide dismutase (SOD) capability. FFM, foxtail millet fermented by *P. geesteranus*; TE, Trolox equivalent; UFM, unfermented foxtail millet. Different letters on the bar charts represent significant differences (*p* < .05).

The in vitro antioxidant capacity of UFM and FFM was evaluated by measuring the scavenging activities of DPPH·, ABTS·^+^, and ·O_2_^−^ radicals, along with the assessment of T‐AOC and SOD activities. DPPH· and ABTS·^+^ are frequently used as stable free radical compounds to evaluate the scavenging ability of antioxidants against free radicals. In the DPPH· antioxidant activity analysis, antioxidants inhibit the DPPH· radical, whereas the ABTS·^+^ antioxidant activity analysis assesses the efficacy of hydrogen‐donating antioxidants against nitrogen radicals (Xiang, Li, et al., 2019). As depicted in Figure 4a, the DPPH· and ABTS·^+^ radical scavenging activities of FFM increased by 202.56% and 48.02%, respectively, potentially due to the increased phenolic compound content resulting from SSF. Similar to previous findings, fermentation significantly increased the radical scavenging activity of grain samples (Đorđević et al., 2010; Jan et al., 2022).

·O_2_^−^ is a common free radical produced during metabolic processes in the body, capable of causing damage to DNA and cell membranes through oxidative reactions. Consistent with our observations for DPPH· and ABTS·^+^ radical scavenging activity, the ·O_2_^−^ scavenging capacity of FFM exhibited a significant increase from 40.91 ± 2.06% to 63.75 ± 1.12% (Figure 4b; *p* < .05). This enhanced ability to quench ·O_2_^−^ indicates that FFM by *P. geesteranus* can more effectively interfere with oxidative chain reactions.

The T‐AOC and SOD antioxidant capacities of foxtail millet flour were also significantly influenced by fermentation (Figure 4c,d), increasing by 2.16 and 1.74 times, respectively. Excessive generation of free radicals beyond the scavenging capacity of the cellular system can lead to oxidative stress, resulting in detrimental effects on cells. T‐AOC and SOD play crucial roles in reducing oxidative stress. T‐AOC can reduce H_2_O_2_ generated during oxidative damage by catalyzing its decomposition, whereas SOD catalyzes the conversion of superoxide radicals to ordinary molecular oxygen (O_2_) or H_2_O_2_ (Reddy & Viswanath, 2019). The in vitro enhancement of the antioxidant properties of FFM flour can be largely attributed to the increase in phenolic compound content (Figure 2) resulting from the action of extracellular enzymes secreted by *P. geesteranus*.

### 3.7 Antioxidant capability of FFM in vivo

As shown in Table S1, the weight gain of mice in group AC was slightly less than those of the other groups after 30 days of continuous feeding; however, there were no significant differences observed in the indicators of liver, heart, and kidney. Additionally, the total feed consumption of all groups exhibited similarity (Table S1). These findings indicate that providing with regular chow diet with vitamin C, UFM flour, or FFM flour had negligible effect on the growth or development of mice in this study.

The T‐AOC, SOD, MDA, and GSH‐Px, key indicators of cellular antioxidant status, were measured in different tissues (Table 2). The T‐AOC indicates the nonenzymatic antioxidant defense system's capacity to counteract oxidative stress. The FFM group exhibited the highest T‐AOC activity in all tissues (Table 2). Compared to the UFM group, T‐AOC in the heart, liver, and kidneys in the FFM group increased by 20.78%, 62.92%, and 53.33%, respectively. Liver tissue exhibited the highest SOD activity, while heart tissue had the lowest (Table 2). Among the four groups, the FFM group exhibited the highest SOD activity in the heart, liver, and kidneys (1.06, 0.58, and 1.06 times of that in UFM, respectively). MDA is a prominent product of lipid oxidation and serves as a reliable marker for assessing oxidative stress. The presence of lipid oxidation products can initiate detrimental oxidative reactions within cell membranes, resulting in cellular dysfunction and an increased risk of various chronic diseases (Dong et al., 2021). Compared to the NC group, the levels of MDA were lower in the PC, UFM, and FFM groups in all tissues. MDA levels in the heart, liver, and kidneys of the FFM group decreased by 11.25%, 23.78%, and 11.93%, respectively, compared with the UFM group. GSH‐Px is a selenium‐dependent enzyme with great significance in preventing the occurrence of cardiovascular diseases (Benstoem et al., 2015). In the tissues tested, a notable increase in GSH‐Px activity was observed, following the trends observed for SOD activity (Table 2). GSH‐Px activity in the heart, liver, and kidneys of the FFM group was 28.60%, 46.79%, and 80.40% higher, respectively, than that of the UFM group. The enhanced in vivo activity of T‐AOC, SOD, and GSH‐Px, along with the reduction of MDA levels, is indicative of effective scavenging of diverse forms of oxygen free radicals and their products. These results indicate that FFM has better antioxidant activity in vivo than UFM. This observation concurs with those of other studies (Balli et al., 2020; Shen et al., 2017), demonstrating that fermentation can enhance the antioxidant properties of grains.

**TABLE 2:** Effect of fermented undehusked foxtail millet on the T‐AOC (U/mg protein), SOD (U/mg protein), and GSH‐Px (U/mg protein) activities and the level of MDA (nmol/mg protein) in different organs in vivo.

Item	NC	PC	UFM	FFM
Heart				
T‐AOC	1.29 ± 0.014^d^	1.76 ± 0.0091^b^	1.54 ± 0.014^c^	1.86 ± 0.017^a^
SOD	21.07 ± 1.09^d^	42.32 ± 2.13^b^	27.17 ± 1.99^c^	56.03 ± 3.08^a^
MDA	3.99 ± 0.099^a^	2.86 ± 0.11^c^	3.11 ± 0.16^b^	2.76 ± 0.13^c^
GSH‐Px	448.9 ± 5.15^d^	576.4 ± 7.02^b^	485.6 ± 4.31^c^	624.5 ± 6.05^a^
Liver				
T‐AOC	1.18 ± 0.062^d^	2.25 ± 0.037^b^	1.53 ± 0.081^c^	5.54 ± 0.16^a^
SOD	151.92 ± 14.19^d^	248.95 ± 13.63^b^	216.89 ± 3.80^c^	342.79 ± 4.31^a^
MDA	3.69 ± 0.36^a^	2.46 ± 0.15^c^	2.86 ± 0.12^b^	2.18 ± 0.06^d^
GSH‐Px	483.81 ± 7.57^d^	554.28 ± 4.18^b^	496.10 ± 2.93^c^	728.21 ± 9.90^a^
Kidneys				
T‐AOC	0.68 ± 0.041^d^	1.73 ± 0.061^b^	0.90 ± 0.027^c^	2.28 ± 0.056^a^
SOD	41.77 ± 0.39^d^	103.4 ± 3.12^b^	64.87 ± 0.89^c^	133.5 ± 7.51^a^
MDA	3.48 ± 0.14^a^	2.45 ± 0.21^c^	2.85 ± 0.13^b^	2.51 ± 0.033^c^
GSH‐Px	314.60 ± 9.36^d^	593.01 ± 7.11^b^	357.40 ± 2.52^c^	644.76 ± 5.46^a^

*Note*: Values represent the means ± SD of three replicates. Different superscript letters in the same row indicate significant differences (*p* < .05).

Abbreviations: FFM, undehusked foxtail millet fermented by *P. geesteranus*; GSH‐Px, glutathione peroxidase.; MDA, malondialdehyde; NC, negative control; PC, positive control; SOD, superoxide dismutase; T‐AOC, total antioxidant capability; UFM, unfermented undehusked foxtail millet.

## 4 CONCLUSIONS

In this study, 15 strains of edible fungi were used for the SSF of FFM. Through screening of the phenolic compound content, antioxidant properties, and anti‐nutrient phytic acid content, *P. geesteranus* was identified as the best candidate for fermentation. By analyzing changes in the phenolic compound content, extracellular enzyme activity, and internal SEM images of undehusked foxtail millet following different fermentation periods, the optimal fermentation period for *P. geesteranus* SSF of undehusked foxtail millet was determined to be 30 days. Under these conditions, FFM exhibited favorable physical and functional properties, elevated nutritional content, and enhanced in vitro and in vivo antioxidant capacities. The bulk density, swelling power, solubility, sedimentation volume ratio, freeze–thaw stability, transparency, oil absorption capacity, and water absorption capacity of FFM were 0.65 ± 0.023 g/mL, 5.96 ± 0.036%, 12.45 ± 0.14%, 3.29 ± 0.41%, 86.42 ± 1.54%, 19.71 ± 1.11%, 2.12 ± 0.061 g/g, and 3.16 ± 0.071 g/g, respectively. The crude protein, crude fiber, phytic acid, vitamin C, crude fat, and crude polysaccharide of FFM were 11.38 ± 0.16 g/100 g, 5.07 ± 0.046 g/100 g, 0.12 ± 0.020 mg/g, 0.92 ± 0.028 mg/100 g, 1.93 ± 0.016 g/100 g, and 0.37 ± 0.0045 mg/100 g, respectively. The scavenging activities of DPPH·, ABTS·^+^, and ·O_2_^−^ radical of FFM in vitro were found to be 73.19%, 93.86%, and 63.75%, respectively, along with T‐AOC and SOD activities increasing by 2.16 and 1.74 times, respectively. The activity of T‐AOC, SOD, and GSH‐Px increased, and the levels of MDA were decreased in vivo in FFM. Our findings provide a reference for the enhanced processing of millet and other grains to produce nutritionally superior products for the feed and food industries.

## AUTHOR CONTRIBUTIONS

**Tong Lin:** Conceptualization (equal); data curation (equal); formal analysis (equal); investigation (equal); methodology (equal); validation (equal); writing – original draft (equal). **Zhanyong Li:** Data curation (equal); methodology (equal). **Gongjian Fan:** Data curation (equal); formal analysis (equal); investigation (equal). **Chunyan Xie:** Conceptualization (equal); investigation (equal); project administration (equal); supervision (equal); writing – review and editing (equal).

## CONFLICT OF INTEREST STATEMENT

There are no conflicts of interest to declare.

## ETHICS STATEMENT

The care and use of laboratory animals reported in this study were approved by the Animal Research Committee of the Langfang Normal University and the Ministry of Agriculture of China (GB/T 35892‐2018).

## Supporting information


Appendix S1.

## Data Availability

All data can be obtained from the corresponding author upon reasonable request.
