# Supplementary material for: Enhancing the nutritional value and antioxidant properties of foxtail millet by solid‐state fermentation with edible fungi
Source: Food Sci Nutr. 2024 Jul 1;12(9):6660–72. doi: 10.1002/fsn3.4203 (PMC11561778; doi:10.1002/fsn3.4203)
Supplement: Supplementary file 1 — Appendix S1. [file FSN3-12-6660-s001.docx]

Supplementary materials

**Enhancing the** **nutritional value and antioxidant properties of foxtail millet by solid state fermentation with edible fungi**

Tong Lin^a,b,c^, Zhanyong Li^a,b,c^, Gongjian Fan^d^ and Chunyan Xie^a,^^b,c^ *

^a^ College of Life Science, Langfang Normal University, Langfang 065000, Hebei, China

^b^ Technical Innovation Center for Utilization of Edible and Medicinal Fungi in Hebei Province, Langfang 065000, Hebei, China

^c^Edible and Medicinal Fungi Research and Development Center of Hebei Universities, Langfang 065000, Hebei, China

^d^College of Light Industry and Food Engineering, Nanjing Forestry University, Nanjing 210037, China P.R., China

* Corresponding author: Chunyan Xie (Email: [1231597@lfnu.edu.cn](mailto:1231597@lfnu.edu.cn))


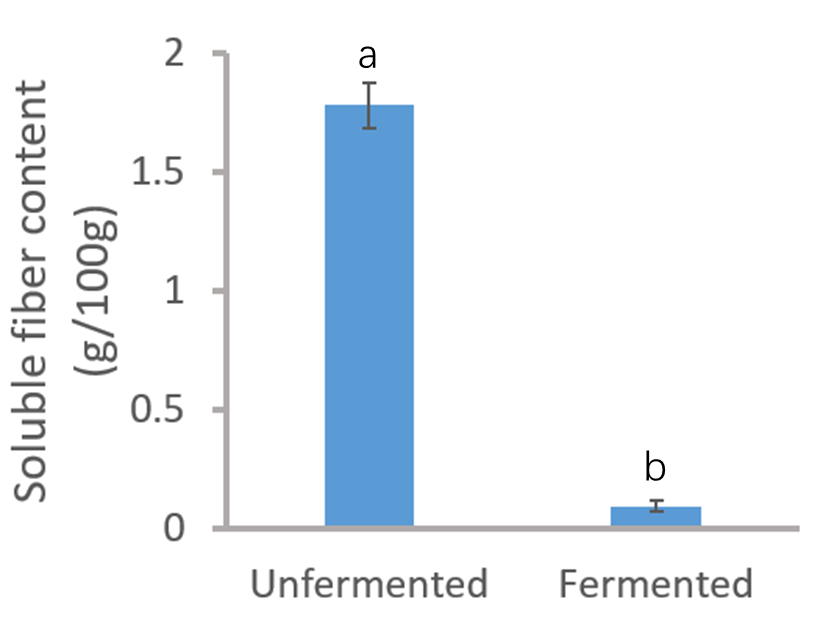


Figure S1. Soluble fiber content in foxtail millet fermented by *P. geesteranus.*


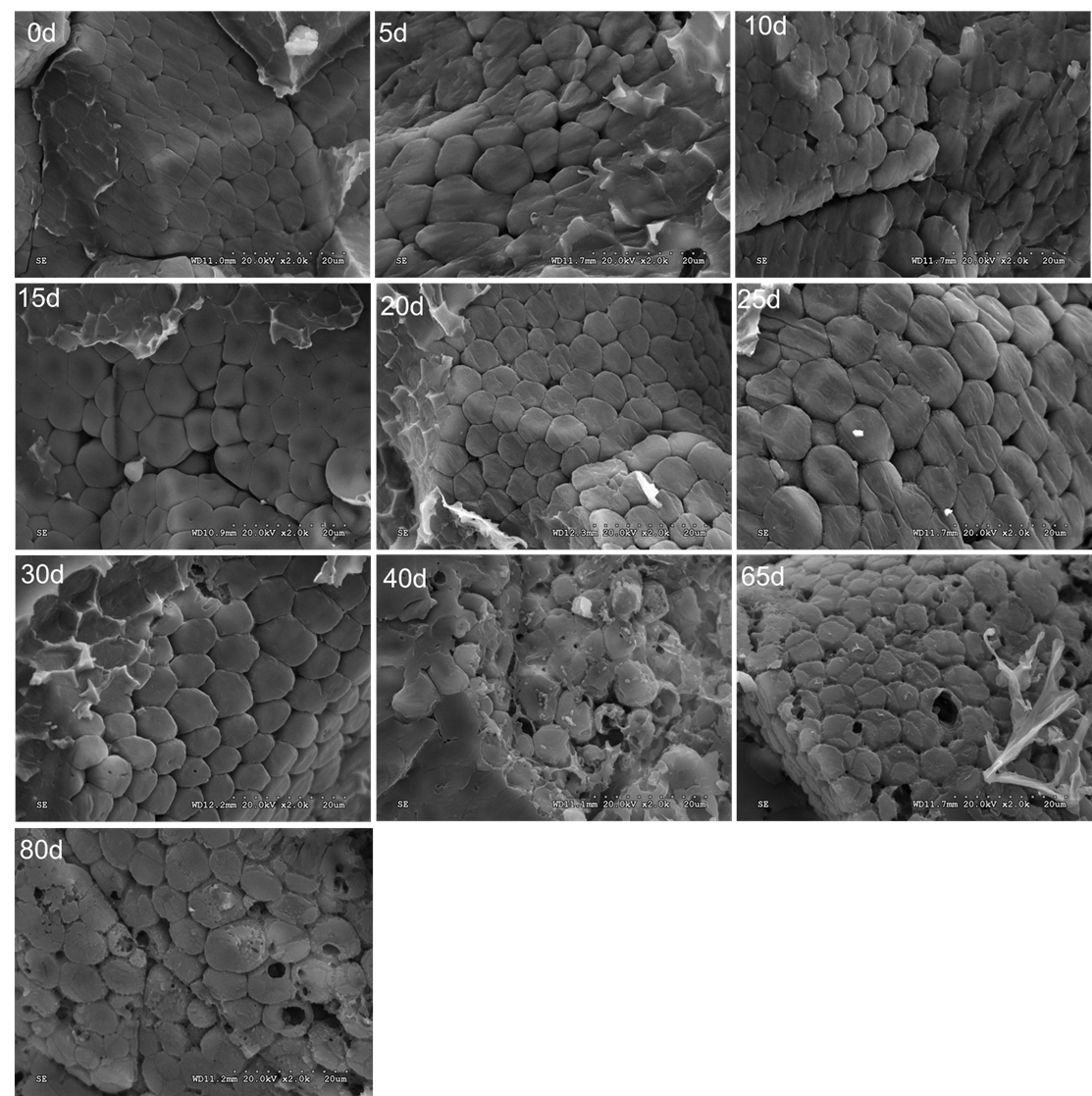


Figure S2. SEM image of the inside of foxtail millet fermented with different times at 20 kV EHT, 12.2 mm WD and 20μm size.

Table S1. Effect of fermented foxtail millet on body weight gain, tissues index and total feed consumption of mice (n=10 per group).

| Groups | Body weight gain/g | Liver index/% | Heart index/% | Kidney index/% | Total feed consumption/g |
| --- | --- | --- | --- | --- | --- |
| AC | 4.82 ± 0.95^b^ | 4.25 ± 0.26^a^ | 0.49 ± 0.019^a^ | 1.65 ± 0.14^a^ | 148.54 ± 4.36^a^ |
| PC | 5.41 ± 2.12^a^ | 4.54 ± 0.48^a^ | 0.51 ± 0.050^a^ | 1.54 ± 0.19^a^ | 145.11 ± 2.19^a^ |
| UFM | 5.01 ± 1.98^a^ | 4.71 ± 0.54^a^ | 0.44 ± 0.021^a^ | 1.71 ± 0.22^a^ | 142.86 ± 3.11^a^ |
| FFM | 4.96 ± 2.05^a^ | 4.39 ± 0.41^a^ | 0.46 ± 0.036^a^ | 1.49 ± 0.30^a^ | 144.08 ± 1.97^a^ |

AC, the naive group provided with a normal chow diet; PC, the positive control group provided with a diet made of a mixture of normal chow and Vitamin C (50 mg/kg body wt.); UFM and FFM were two test groups provided with a diet made of a mixture of normal chow and foxtail millets and a mixture of normal chow and fermented foxtail millets, respectively, with all the millets contents of 200 g/kg. All the diets were processed by SPE Biotechnology Co., Ltd. Results are presented as the mean (n = 10) ± standard deviation. Values carrying different letters in the same column are significantly different (*P* < 0.05).
